# Donor Haplotype B of NK KIR Receptor Reduces the Relapse Risk in HLA-Identical Sibling Hematopoietic Stem Cell Transplantation of AML Patients

**DOI:** 10.3389/fimmu.2014.00405

**Published:** 2014-08-25

**Authors:** Ulla Impola, Hannu Turpeinen, Noora Alakulppi, Tiina Linjama, Liisa Volin, Riitta Niittyvuopio, Jukka Partanen, Satu Koskela

**Affiliations:** ^1^FRC Blood Service, Research and Development, Helsinki, Finland; ^2^Institute of Biosciences and Medical Technology (BioMediTech), University of Tampere, Tampere, Finland; ^3^Division of Hematology, Helsinki University Central Hospital, Helsinki, Finland

**Keywords:** NK cells, KIR, HLA, graft versus tumor effect, transplantation immunology

## Abstract

Successful allogeneic hematopoietic stem cell transplantation (HSCT) depends not only on good HLA match but also on T-cell mediated graft-versus-leukemia (GvL) effect. Natural killer (NK) cells are able to kill malignant cells by receiving activation signal from the killer-cell immunoglobulin-like receptors (KIR) recognizing HLA molecules on a cancer cell. It has been recently reported that the risk of relapse in allogeneic hematopoietic stem cell transplantation (HSCT) is reduced in acute myeloid leukemia (AML) patients whose donors have several activating KIR genes or KIR B-motifs in unrelated donor setting, obviously due to enhanced GvL effect by NK cells. We studied the effect on relapse rate of donor KIR haplotypes in the HLA-identical adult sibling HSCT, done in a single center, in Helsinki University Central Hospital, Helsinki, Finland. Altogether, 134 patients with 6 different diagnoses were identified. Their donors were KIR genotyped using the Luminex and the SSP techniques. The clinical endpoint, that is, occurrence of relapse, was compared with the presence or absence of single KIR genes. Also, time from transplantation to relapse was analyzed. The patients with AML whose donors have KIR2DL2 or KIR2DS2 had statistically significantly longer relapse-free survival (*P* = 0.015). Our data support previous reports that donors with KIR B-haplotype defining genes have a lower occurrence of relapse in HSCT of AML patients. Determination of donor KIR haplotypes could be a useful addition for a risk assessment of HSCT especially in AML patients.

## Introduction

Allogeneic hematopoietic stem cell transplantation (HSCT) is an established curative treatment for many hematological malignancies. The outcome of HSCT is strongly influenced by the genetic differences between donor/recipient pairs (1). Genetic similarity or identity in the HLA genes in the major histocompatibility complex on chromosome 6, affects the incidence of graft-versus-host disease (GvHD), the major complication of HSCT. Successful allogeneic HSCT, however, depends also on T-cell mediated graft-versus-leukemia (GvL) effect, in which donor-derived T cells clear the remaining leukemic cells in patient. In addition to alloreactive T cells, donor-derived natural killer (NK) cells, are able to kill these malignant or virus-infected cells in the patient. NK cells might therefore have a crucial role in relapse prevention by destroying remaining acute myeloblastic leukemia cells (2, 3).

Natural killer cells are regulated by their surface receptors, which have either activating or inhibitory function by interacting with MHC class I molecules, i.e., group 1 and group 2 HLA-C alleles and Bw4 alleles, present on normal cell surface. Activation occur when the NK cell receives a signal from the killer-cell immunoglobulin-like receptors (KIR) that recognize affected or tumor cell lacking the normal HLA structures on its surface. The ratio of activating and inhibitory receptors on the NK cell surface determines how easily NK cell is activated (4).

**Figure 1:**
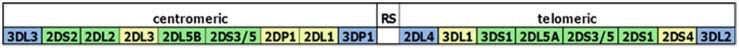
**The KIR locus in chromosome 19 is part of the leukocyte receptor complex (LRC) region and is composed of centromeric and telomeric parts**. The two parts are separated by a recombination site (RS) sequence. B-haplotype defining genes are depicted in green color, and A-haplotype defining genes are depicted in yellow color. The framework genes that are present in each haplotype are blue.

Killer-cell immunoglobulin-like receptors are genetically highly polymorphic and are expressed in a stochastic manner. The HLA and KIR genes are located in the different chromosomes and are therefore inherited independently. Consequently, many people may have activating and/or inhibitory KIR genes for which they do not have the HLA ligands. Only minority of HLA-matched donor-patient pairs have the same KIR haplotypes and in case of HLA-matched siblings, KIR haplotype identity occurs in 25% of donor-patient pairs (5). Seventeen of the KIR genes can be categorized for the inhibitory (KIR A-haplotype) or the activating haplotypes (KIR B haplotype) based on their gene content (6). These haplotypes can be further divided as telomeric and centromeric parts, which contain either A- or B-motifs according to the presence or absence of A- or B-haplotype defining KIR genes (Figure 1).

The first evidence of the role of the KIR genes in HSCT came from haploidentical transplantation studies by Ruggeri et al. They showed that acute myeloid leukemia (AML) patients benefit from the KIR-ligand mismatch due to the “missing self” phenomenon (7, 8). In addition, there is evidence that the risk of relapse is reduced in those AML patients whose donors have several activating KIR genes or KIR-B gene-motifs in unrelated donor (URD) HSCT (9, 10). However, there are also some conflicting results (10–12). Clinical benefits have been speculated to be gained in settings, where donor NK cells have at least one inhibitory KIR gene for which they lack the specific HLA class I ligand (13).

In the present study, we looked if activating KIR genes in the HLA-matched related donors (RD) have any effect on relapse incidence or rate in transplanted patients. Altogether, 134 HLA-matched related sibling pairs were included in this study. We further focused the AML patients and their donors as the number of patients with other diagnosis was low and the most promising results about KIR genes or KIR ligands have been observed in the AML patients. The clinical data were compared with the presence or absence of single KIR genes and with the KIR B-content score. Our results support previous findings that donors with KIR B-haplotype defining genes contribute the GvL effect resulting in a lower relapse incidence in AML patients after HSCT.

## Materials and Methods

### Patients

Altogether, 134 adult patients who had undergone related donor HSCT and their donors were included into this study. Patient cohort consisted of 6 different diagnoses; 47 patients had AML (Table 1). Transplantations were performed in Helsinki University Central Hospital Hematology Clinic, Helsinki, Finland, during 1993–2004. The set of patients is basically identical to that described in our previous studies (14, 15). Informed consent was obtained from all subjects and the study protocol was approved by the local Ethical Review Board of the Helsinki University Hospital, Helsinki, Finland.

**Table 1:** **Altogether, 134 patients with 6 diagnoses were included in statistical analysis**.

Diagnosis	No. of patients	Relapse	aGvHD grade II–IV	Graft type		Disease status	
				PB	BM	Good prognosis	Bad prognosis
AML	47	14	11	15	32	33	14
MDS	16	5	2	3	13	6	10
CML	31	9	10	7	24	20	11
CLL/NHL	13	1	2	5	8	9	4
ALL	27	8	5	7	20	19	8
Total	134	37	30	37	97	87	47

*Diagnoses were: acute myeloid leukemia (AML), myelodysplastic syndrome (MDS), chronic myeloid leukemia (CML), chronic lymphoid leukemia (CLL), non-Hodgkin lymphoma (NHL), acute lymphoid leukemia ALL. Graft types used were peripheral blood (PB) or bone marrow (BM)*.

### Genotyping

#### HLA

All patients and donors were typed for HLA-A, -B, -C, -DRB1, and -DQB1 using EFI accredited typing methods in routine typing of the transplantation candidates either by serological method (Lymphotype HLA-AB and Lymphotype HLA-DR-DQ, Bio-Rad Medical Diagnostics, Dreieich, Germany) or by using PCR-based typing methods using the LIPA HLA-C reverse dot blot kit (Innogenetics Group, Gent, Belgium), or the Pel Freez HLA-C SSP kits (Dynal Biotech LLC, Oslo, Norway), or the LABType SSO HLA typing Test (One Lambda Inc., 21001 Kittridge Street, Canoga Park, CA 91303-2801, USA). Only 10/10 HLA-matched siblings were included in the study.

#### KIR genotyping

Samples were whole genome amplified by using the GenomiPhi V2 DNA Amplification Kit (GE Healthcare UK Ltd., Chalfont, Bucks, UK). The amplified DNA samples from the donors were KIR genotyped by using the LABType SSO KIR typing Test (One Lambda Inc., 21001 Kittridge Street, Canoga Park, CA 91303-2801, USA) according to the manufacturer’s instructions or in some cases by using Olerup SSP^®^ KIR Genotyping kit (Olerup SSP AB, Stockholm, Sweden).

### Statistical analysis

The incidence of the relapse was compared with the presence or absence of single KIR genes and with the KIR B-content score (IPD KIR sequence database, EBI, B-content calculator) within each diagnosis category. The Log-rank (Peto) and the Wilcoxon tests were used for the statistical analysis by using the StatsDirect software (StatsDirect Ltd. 9 Bonville Chase, Altrincham Cheshire, UK).

Also, time to relapse was analyzed in survival analyses using Kaplan–Meier and Cox regression analyses.

## Results

In this study, we looked if activating KIR genes in the HLA-matched RD have any effect on relapse rate in transplanted patients. The KIR-B-content score and single KIR genes were evaluated against relapse both in the whole patient cohort and within each diagnosis category.

**Figure 2:**
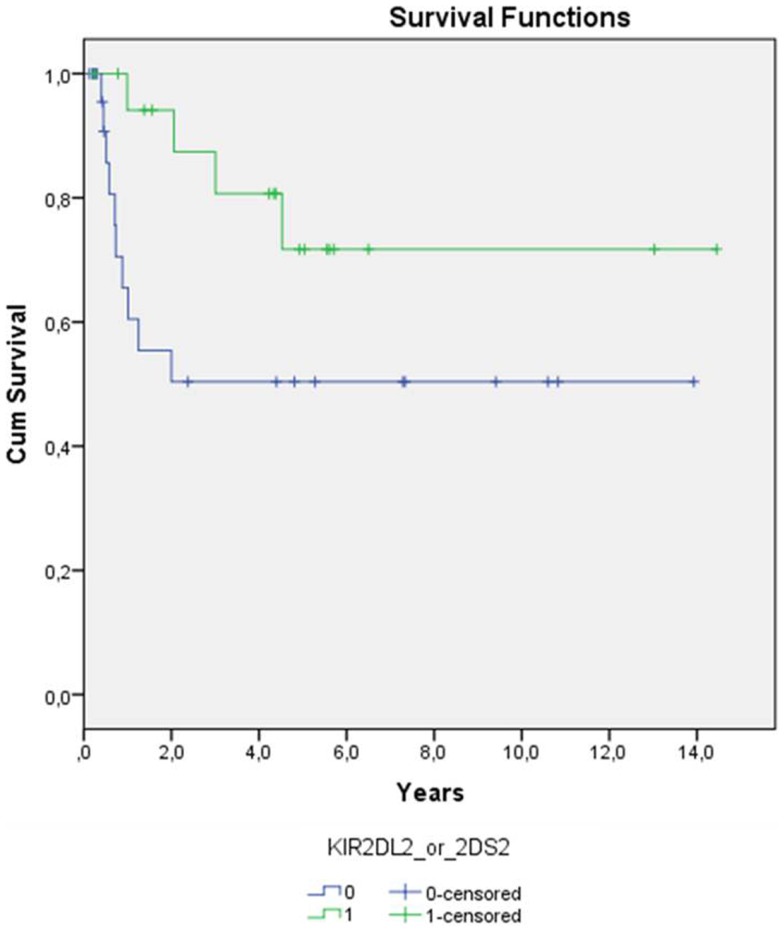
**Time from transplantation to relapse among patients with AML (*n* = 47) is shown**. Patients whose donors have either KIR2DL2 or KIR2DS2 (*n* = 19, green line) have lower risk for relapse (log-rank *p*-value 0.059) than patients whose donors do not have those B haplotype defining KIR genes (*n* = 28, blue line).

Statistical significance for better relapse-free survival (log-rank *p*-value of 0.059 in Kaplan–Meier) was observed for AML patients whose donor had KIR2DS2 or KIR2DL2 (Figure 2). This effect was even strengthened when other factors affecting relapse (a/cGvHD, graft type, disease status, GvHD prevention) were included in the analyses (Cox regression *p* = 0.015, Table 2). Graft type and disease status also had an effect on the relapse rate as expected (*p* = 0.050 and 0.014, respectively). Longer relapse-free survival was observed in the patients receiving peripheral blood stem cells compared to the patients receiving bone marrow and also in the patients with good prognosis compared to the patients with bad prognosis. No significant *P*-values were observed for other KIR genes or diagnoses.

**Table 2:** **Multivariate Cox regression analyses of relapse rate in AML patients**.

	B	SE	Wald	df	Sig.	Exp(B)	95.0% CI for Exp(B)	
							Lower	Upper
KIR2DL2_or_2DS2	−2.038	0.838	5.917	1	0.015	0.130	0.025	0.673
aGvHD	0.141	0.859	0.027	1	0.870	1.151	0.214	6.196
cGvHD	−0.005	0.117	0.002	1	0.968	0.995	0.791	1.253
Graft_type	1.718	0.878	3.831	1	0.050	5.575	0.998	31.150
GvHD prevention	1.214	1.152	1.110	1	0.292	3.366	0.352	32.210
Disease status	2.299	0.937	6.022	1	0.014	9.968	1.589	62.546

*Presence of donor KIR2DL2 and KIR2DS2 as well as good prognosis and using bone marrow as stem cell source has a beneficial influence on patients’ relapse-free survival*.

Statistical significance was not reached when the donor KIR-B-content score was evaluated in AML patients, but a trend between high KIR-B-content score and low relapse rate could be seen (Log-rank *P* = 0.39, Wilcoxon *P* = 0.39, data not shown).

If all the patients with different diagnoses were included in the tests, the statistical significance or trend was lost both in single gene analysis and B-content score analysis. The frequencies of KIR genes in the donors were similar to those reported earlier (16).

## Discussion

HLA matching is a prerequisite for the successful HSCT and is the most important factor that is taken into account before transplantation. There is increasing evidence that also other genes of the genome like KIR genes and minor histocompatibility antigens (mHAs) (17–19) contribute to the outcome. The effect of the KIR genes has been studied in different settings where either related or URDs were used for either T-cell depleted or repleted patients (7, 9, 20–22).

Ruggeri et al. demonstrated an advantageous effect on the survival of the AML patients in the KIR-ligand mismatched haploidentical transplantation in which the donor NK cell recognition of the missing self on the recipient targets is associated with the GvL effect. The positive effect on the survival of the AML patients was also observed in URD transplantations when the KIR genes were grouped according to the number of the activating and inhibitory KIR genes. Donors with more activating KIR genes (KIR B-haplotype) had more GvL effect than the donors with inhibitory KIR genes (KIR A-haplotype). The greater the KIR B-score was, the less relapses were observed (9). As there are as many as 17 KIR genes that form different haplotypes and as each of them has the different ligand specificity, the mechanism how these parameters act together in a beneficial manner must be complex.

In this study, donor KIR2DS2 and KIR2DL2 that are KIR B-haplotype defining genes, were associated with the longer relapse-free survival of AML patients. It seems that the donors with KIR B-haplotype defining genes may protect patients against relapse especially in myeloblastic leukemias compared to the donors without or with only few activating KIR genes. The enhanced GvL effect was even strengthened when other relapse-affecting factors such as graft type and disease status were included in multivariate analyses. As a/cGvHD or GvHD prevention did not seem to affect the incidence of relapse, it is possible that biological agents that are involved in these phenomena are different from the GvL. These results together with Cooley et al. suggest that selecting donors with KIR-B haplotype defining genes may be beneficial in different transplantation settings since our study comprised fully matched RD and Cooley et al. studied registry donors. In addition, there is evidence that donors with several activating KIR genes protect patients from the CMV activation after HSCT (23–25) although in some studies no beneficial effect was observed (10, 26).

It must be noted that the *P* values in our study are influenced by the small sample size as 134 patients represented 6 different diagnoses and several different KIR genes exist in human genome and therefore studies with larger cohort are needed. Our results support, however, the observation that selecting the donors with more KIR B-haplotype defining genes may lower the risk of relapse in HSCT. Taking the KIR genotype or KIR-ligand mismatch into account in donor selection may have a beneficial effect on the patients’ survival and at least is a valuable tool in risk assessment in any transplantation setting. However, the downside of the selecting donors with high KIR B-content score and/or appropriate KIR ligand is the increased incidence of the GvHD, the life threatening condition (27, 28).

## Author Contributions

Satu Koskela, Ulla Impola, Hannu Turpeinen, Noora Alakulppi, Tiina Linjama, and Jukka Partanen designed this study, performed the experimental design and data analysis. They also provided their intellectual content for the study. Liisa Volin and Riitta Niittyvuopio provided patient cohort and clinical data integration. Satu Koskela, Ulla Impola, Hannu Turpeinen, and Jukka Partanen wrote the manuscript.

## Conflict of Interest Statement

The authors declare that the research was conducted in the absence of any commercial or financial relationships that could be construed as a potential conflict of interest.
